# Delayed Motor Development and Infant Obesity as Risk Factors for Severe Deformational Plagiocephaly: A Matched Case–Control Study

**DOI:** 10.3389/fped.2020.582360

**Published:** 2020-11-11

**Authors:** Eun-Hee Kim, Ki Eun Kim, Jihyun Jeon, Youn Ho Sheen, Hyun-Seung Lee, So Young Yoon, Nam Hyo Kim, Kyoung Min Choi

**Affiliations:** ^1^Department of Pediatrics, Sejong Chungnam National University Hospital, Chungnam National University School of Medicine, Sejong, South Korea; ^2^Department of Pediatrics, CHA Gangnam Medical Center, CHA University School of Medicine, Seoul, South Korea

**Keywords:** deformational plagiocephaly, risk factors, development, obesity, tummy time

## Abstract

The prevalence of deformational plagiocephaly (DP) has increased since the recommendation of positioning infants to their back during sleeping and is affected by various biological and environmental factors. This study aimed to investigate associations between DP and perinatal or infant characteristics, including obesity. This case–control study included 135 infants (81 males) aged 2–12 months who were diagnosed with DP using calculated cranial vault asymmetric index and cranial index and 135 age- and sex-matched controls. Motor development was evaluated using the Alberta Infant Motor Scale, and obesity was defined by body mass index. Univariate and multivariate logistic regression models were used to assess potential risk factors for DP and its severity. One hundred thirty-five infants with DP were divided into the following three subgroups according to severity indicated by the cranial vault asymmetry index: mild to moderate group (*n* = 87, 64.4%), severe group (*n* = 48, 35.6%), and a combined plagiocephaly and brachycephaly group (*n* = 79, 58.5%). Independent risk factors significantly associated with development of DP were bottle-only feeding (adjusted odds ratio (aOR) = 4.65; 95% CI: 2.70–8.00), little tummy time when awake (aOR = 3.51, 95% CI: 1.71–7.21), delay of motor development (aOR = 2.85, 95% CI: 1.08–7.49), and obesity at diagnosis (aOR = 2.45, 95% CI: 1.02–5.90). Among these risk factors, delay of motor development (aOR = 4.91, 95% CI: 1.46–16.51) and obesity at diagnosis (aOR = 4.10, 95% CI: 1.42–11.90) were particularly related to severe DP. In conclusion, this study confirms that DP risk is positively associated with bottle-only feeding, infrequent tummy time, and delayed development of motor milestones. Notably, this study demonstrates infant obesity as a new risk factor for DP. Our findings suggest that obesity should be identified early and managed comprehensively in infants with DP.

## Introduction

Deformational plagiocephaly (DP) is an asymmetry of the skull caused by external pressure on an infant's head in the absence of premature craniosynostosis (1–3). The prevalence of DP has increased since the 1990s when the American Academy of Pediatrics issued a “Back to Sleep” recommendation to reduce the risk of sudden infant death syndrome, and DP occurs in about 38–47% of infants at 3–4 months of age (4–6). In recent years, due to the initiation of the national health screening system for infants and children, the diagnosis of DP has further increased, and as cranial remodeling orthotic treatment becomes popular, parents' interest in treating DP has also increased (7).

Deformational plagiocephaly develops in infants aged 6–8 weeks, whose heads grow rapidly while lying down, and this is analogous to how a pumpkin flattens as it grows on the ground (“Pumpkin Analogy”) (1). Later, DP can be improved naturally as infants grow and corrected early by counter-positioning or physiotherapy (8–10). However, infants with severe deformation aged 4–6 months and those with deformation without improvement after 6 months of age may optionally require cranial remodeling orthotic treatment before 12 months of age, and this treatment is considerably expensive and lengthy and requires continuous care, which can be a burden to parents (11). In addition, some infants with severe DP are known to have facial asymmetry, ear misalignment, positional torticollis, or neurodevelopment problems, such as delayed motor development and cognitive learning disorder (3, 12–14), suggesting that DP is not just a cosmetic issue but may be associated with increased risk of neurodevelopment problems (15–17). Therefore, it is important to detect DP early in childhood and identify associated factors to prevent and manage DP.

The prevalence of DP increases dramatically in the first 2 months after birth and is affected by various biological and environmental factors (2, 18–20). Proposed biological risk factors for DP include male, torticollis, preferred head position or orientation, higher birth weight, macrocephaly at birth, lower level of activity, and developmental delays (21, 22). Obstetric factors, such as birth order, mode of delivery, prematurity, intrauterine position, multiple birth, or oligohydramnios, are also reported as potential risk factors for DP, although the results of these studies are inconsistent (21, 23, 24). In addition, infant care practice is a risk factor strongly related to the development of DP and includes not varying positions during daily tasks (feeding, asleep, awake, or bottle feeding) and infrequent tummy time. Although many risk factors for DP have been investigated, the findings differ depending on the study cohort and design (2, 19, 23). Indeed, differences in culture-specific beliefs and caregiving practices between families would likely promote different risk factors (19, 25). Although many parents are aware of the need for a supine sleep position in early infants, they are unaware of the need for varying positions or promoting activities, such as tummy time, when infants are awake. In particular, because tummy time is an important physical activity that can prevent cranial deformation, stimulate motor development, and reduce obesity, the lack of tummy time, cranial deformation, obesity, and developmental delay have close associations with each other and should be managed concurrently (16, 26). Studies have investigated the association of tummy time and delayed motor development with DP; however, there are little data on the association between DP and infant obesity. Therefore, this age- and sex-matched case–control study of infants with DP aimed to determine the risk factors for DP and its severity, as well as the association between DP and infant obesity as defined by body mass index (BMI).

## Materials and Methods

### Participants

This study was approved by the CHA Gangnam Medical Center Institutional Review Board (GCI 2020-05-010). Informed consent was not required for this study because it is practically impossible to obtain consent during the retrospective study. Request for waiver of informed consent was approved by the ethics committee due to the retrospective nature of the study. Participants included 135 infants with DP and 135 age- and sex-matched controls enrolled between June 1, 2016 and May 30, 2020 from the CHA Gangnam Medical Center. Inclusion in the study was based on the following criteria: (1) infants were between 2 and 12 months old at the time of diagnosis of DP, (2) cranial index (CI) and cranial vault asymmetry index (CVAI) were assessed using a three-dimensional digital scanner or caliper measurements, and (3) neuroimaging results were negative for craniosynostosis. Any infants with insufficient clinical data, congenital muscular torticollis, congenital anomalies such as craniofacial or chromosomal anomalies, or any birth injury were excluded. Age- and sex-matched controls were randomly selected from the national health screening system for infants and children, and they were confirmed to have no cranial deformation on physical examination.

### Case Definition

In this study, all cases were diagnosed with positional, deformational, or non-synostotic plagiocephaly by a health care provider (E-HK), and each case was assigned a cranial vault asymmetry (CVA) value and a CI value to diagnose head deformation and assess its severity. The CVA, which defines the degree of asymmetry, is the difference in the cranial diagonals between the two sides. The CVAI is computed by dividing the total CVA by the shorter diagonal distance and then multiplying by 100 to be reported as a percentage (27). The CI, which defines the broadness of head proportion, is defined as head width divided by head length, and then multiplied by 100 and is reported as a percentage. Deformational plagiocephaly was diagnosed when the CVA was >10 mm and the CVAI was >3.5% (1, 8). The severity of DP was categorized as mild, moderate, or severe based on the validated Children's Healthcare of Atlanta (CHOA) scale (28). Mild DP was defined as a CVAI between 3.5 and 6.25, moderate was defined as a CVAI between 6.25 and 8.75, and severe was defined as a CVAI >8.75 (28). Although the DP head shape has been described using the CHOA scale, no uniform scale exists for brachycephalic head shapes and a combination of the two deformations. Based on a variety of published scales in the literature and the author's clinical expertise, DP combined with brachycephaly was diagnosed when the CI was >90% (29, 30). All cranial measurements were obtained using a three-dimensional digital scanner (STARscanner; Orthomerica Orlando, FL, USA), and anthropometric measurements were obtained with calipers by a sole experienced pediatric neurologist (E-HK).

### Measures

To investigate the associated factors for DP, we obtained perinatal and infant data from the electronic medical records and selected potential risk factors based on the literature review (2, 17). Clinical data of perinatal factors, including gestational age, weight, and head circumference at birth, multiple births, delivery mode, maternal age, maternal parity, presentation at birth, the presence of cephalopelvic disproportion, oligohydramnios, uterine malformations, and prolonged labor, were collected. Infant factors included sex, age at diagnosis, weight, height, head circumferences at diagnosis, types of feeding (breast feeding, mixed feeding, and only bottle feeding) between 2 and 6 months of age, frequency of tummy time (>5 min) when awake per day before roll-over, and a delay in motor development at diagnosis. To assess obesity at diagnosis, BMI (kg/m^2^) was calculated using weight and height, and BMI z-score and percentile were calculated using sex-specific values according to the 2006 WHO Child Growth Standards with WHO Anthro 2009 Software (31). Specific BMI percentile cutoffs to define children aged ≤2 years as overweight or obese have yet to be agreed upon. However, based on the general recommendation from the WHO charts and published scales in the literature, cutoffs were defined as follows: overweight was defined as BMI z-score > +1 SD (BMI > 85th percentile); obesity as BMI z-score > +2 SD (BMI > 97th percentile) (26, 32–34); normal weight as −1 SD ≤ BMI z-score ≤+1 SD (15th percentile ≤ BMI ≤ 85th percentile); underweight as BMI z-score <-1 SD (BMI <15th percentile) (26, 32–34). Information about tummy time was obtained through interviews, and “little” tummy time was defined as tummy time of 5 min or more but <3 times per day (11). Motor development was assessed through physical examination, and the Alberta Infant Motor Scale (AIMS), a highly reliable and valid measure, was used to examine the spontaneous qualitative gross motor movement repertoire of infants in supine, prone, sitting, and standing positions (35). When the AIMS z-score was <-1 SD, the infant was classified as having delayed motor development (35). Factors related to DP, including the side of a flat spot, the presence of positional torticollis, facial asymmetry and brachycephaly, method of measurement, measured fronto-occipital diameter, biparietal diameter, and the transcranial diagonals (frontotemporal-lambdoid), were collected. Positional torticollis was defined as the head tilt caused by a positional preference of the head without evidence of morphological changes in the neck muscle (36).

### Statistical Analysis

Categorical data are presented as values and percentages, and continuous data as mean and SD. Perinatal and infant factors were analyzed in all infants with DP compared with controls. Infants in each subgroup (mild to moderate DP or severe DP) were compared with controls, and infants with severe DP were compared with those with mild to moderate DP, first by univariate model and then by multivariate model. Univariable analysis was performed to assess risk factors for all DP, mild to moderate DP, and severe DP, and the associations between potential risk factors and each group were shown by odds ratio (OR) and 95% CI. Multivariable logistic regression analysis was performed to minimize the confounding effects by adjusting for potential confounders identified in the univariate analysis and to identify independent risk factors for all, mild to moderate, and severe DP cases. Group differences of continuous variables were assessed using paired *t*-test, independent-samples *t*-test, and one-way ANOVAs. A *p*-value <0.05 was considered statistically significant. All data were analyzed using IBM SPSS 26.0 (Armonk, NY, USA).

## Results

### Clinical Characteristics of Infants With Deformational Plagiocephaly

In total, 135 infants with DP participated in this study (81 males and 54 females), and the mean age at diagnosis was 5.3 ± 1.8 months. Of the 135 infants, 56 (41.5%) had only plagiocephaly, and 79 (58.5%) had a combination of plagiocephaly and brachycephaly. The posterior flat side was on the right in 90 infants (66.7%) and on the left in 45 infants (33.3%). Positional torticollis was identified in 26 infants (19.3%).

One hundred thirty-five infants with DP were divided into the following two subgroups according to the severity indicated by the CVAI: mild to moderate group (*n* = 87, 64.4%) and severe group (*n* = 48, 35.6%).

### Risk Factors for Deformational Plagiocephaly

Perinatal and infant characteristics of cases and controls are summarized in Table 1. Compared with controls, infants with DP were more likely to have only been bottle-fed (*p* < 0.001), have little tummy time when awake (*p* < 0.001), be obese at the time of diagnosis (BMI > 97th percentile) (*p* = 0.007), and present with delayed motor development (*p* = 0.044). However, previously proposed risk factors such as history of prematurity, large for gestational age, macrocephaly at birth, maternal history of vaginal delivery, multiple births, primiparity, and macrocephaly at the time of diagnosis were not significantly different between infants with DP and controls. Moreover, we found no difference between infants with DP and controls in mean values of gestational age, singleton birth weight, maternal age, and BMI at diagnosis.

**Table 1:** Clinical profile of cases and matched controls and odds ratios (ORs) for all, mild to moderate, and severe deformational plagiocephaly (DP) cases.

	**Controls (*n* = 135)**	**Cases (*n* = 135)**	**Univariate analysis**^**a**^		**Mild to moderate DP (*n* = 87)**	**Univariate analysis**^**b**^		**Severe DP (*n* = 48)**	**Univariate analysis**^**c**^	
			***p*-values**	**OR (95% CI)**		***p*-values**	**OR (95% CI)**		***p*-values**	**OR (95% CI)**
Male, *n* (%)	81 (60.0)	81 (60.0)	Matched		52 (59.8)	0.973	0.99 (0.57–1.72)	29 (60.4)	0.960	1.02 (0.52–2.00)
Age at diagnosis, months, mean ± SD	5.2 ± 1.9	5.3 ± 1.8	Matched		5.3 ± 1.6	0.836		5.3 ± 2.1	0.824	
**Perinatal variables**										
Gestational age, weeks, mean ± SD	38.5 ± 1.8	38.4 ± 1.6	0.560		38.4 ± 1.6	0.655		38.3 ± 1.7	0.625	
Prematurity, *n* (%)	16 (11.9)	23 (17.0)	0.240	1.52 (0.76–3.02)	16 (18.4)	0.186	1.66 (0.78–3.53)	7 (14.6)	0.637	1.26 (0.48–3.28)
Singleton birth weight, g, mean ± SD	3,149.1 ± 428.3	3,126.9 ± 443.3	0.697		3,042.5 ± 503.1	0.808		3,066.5 ± 434.2	0.929	
LGA, *n* (%)	15 (11.1)	11 (8.1)	0.410	0.71 (0.31–1.61)	7 (8.0)	0.457	0.70 (0.27–1.79)	4 (8.3)	0.589	0.73 (0.23–2.31)
Maternal age, years, mean ± SD	34.9 ± 3.5	34.5 ± 3.8	0.536		34.5 ± 3.4	0.552		34.6 ± 4.8	0.755	
Primiparity, *n* (%)	122 (90.4)	114 (84.4)	0.146	0.58 (0.28–1.21)	75 (86.2)	0.340	0.67 (0.29–1.54)	39 (81.3)	0.101	0.46 (0.18–1.16)
Vaginal delivery, *n* (%)	53 (39.3)	63 (46.7)	0.220	1.35 (0.84–2.20)	40 (46.0)	0.753	1.09 (0.63–1.89)	23 (47.9)	0.297	1.42 (0.73–2.76)
Multiple birth, *n* (%)	18 (13.4)	15 (11.1)	0.560	0.81 (0.39–1.67)	12 (13.8)	0.939	1.03 (0.47–2.26)	3 (6.3)	0.192	0.43 (0.12–1.53)
**Infant variables at diagnosis**										
Only bottle feeding, *n* (%)	50 (37.0)	97 (71.9)	** <0.001**	4.34 (2.60–7.25)	63 (72.4)	** <0.001**	4.46 (2.48–8.02)	34 (70.8)	** <0.001**	4.13 (2.02–8.43)
Little tummy timewhen awake, *n* (%)	95 (70.4)	121 (89.6)	** <0.001**	3.64 (1.87–7.08)	77 (88.5)	**0.002**	3.24 (1.52–6.90)	44 (91.7)	**0.006**	4.63 (1.56–13.75)
BMI (kg/m^2^) at diagnosis, mean ± SD	17.8 ± 1.4	18.1 ± 1.8	0.157		18.1 ± 1.8	0.139		18.0 ± 1.8	0.392	
Underweight, *n* (%)	4 (3.0)	7 (5.2)	0.271	2.03 (0.58–7.20)	6 (6.9)	0.125	2.79 (0.75–10.35)	1 (2.1)	0.823	0.78 (0.08–7.21)
Normal weight, *n* (%)	93 (68.9)	80 (59.3)			50 (57.5)	0.068		30 (62.5)	0.063	
Overweight, *n* (%)	29 (21.5)	24 (17.8)	0.902	0.96 (0.52–1.79)	19 (21.8)	0.806	1.09 (0.55–2.18)	5 (10.4)	0.235	0.53 (0.19–1.508)
Obese, *n* (%)	9 (6.7)	24 (17.8)	**0.007**	3.10 (1.36–7.06)	12 (13.8)	**0.021**	2.89 (1.17–7.15)	12 (25.0)	**0.004**	4.13 (1.59–10.77)
Macrocephaly, *n* (%)	12 (8.9)	17 (12.6)	0.426	1.38 (0.63–3.04)	12 (13.8)	0.372	1.48 (0.62–3.53)	5 (10.4)	0.754	1.19 (0.40–3.58)
Delayed motor development, *n* (%)	8 (5.9)	18 (13.3)	**0.044**	2.44 (1.02–5.83)	9 (10.3)	0.232	1.83 (0.68–4.95)	9 (18.8)	**0.012**	3.66 (1.32–10.14)

*BMI, body mass index; DP, deformational plagiocephaly; LGA, large for gestational age; OR, odds ratio*.

a *Univariable logistic regression analyses of risk factors associated with deformational plagiocephaly*.

b *Univariable logistic regression analyses of risk factors associated with mild to moderate deformational plagiocephaly*.

c *Univariable logistic regression analyses of risk factors associated with severe deformational plagiocephaly*.

*Significant P-values (<0.05) are indicated in bold*.

Multivariate analysis found that DP was independently associated with only bottle feeding (adjusted odds ratio (aOR) = 4.65; 95% CI: 2.70–8.00) and little tummy time when awake (aOR = 3.51, 95% CI: 1.71–7.21). In addition, a 2-fold increase in DP risk was observed in individuals with delayed motor development (aOR = 2.85, 95% CI: 1.08–7.49) and obesity at diagnosis (aOR = 2.45, 95% CI: 1.02–5.90) (Table 2).

**Table 2:** Multivariable analysis on the association between clinical variables and all, mild to moderate, and severe deformational plagiocephaly (DP) cases.

**Independent risk factors**	**Adjusted OR**	**95% CI**	***p*-values**
**For DP**			
Only bottle feeding	4.65	2.70–8.00	<0.001
Little tummy time when awake	3.51	1.71–7.21	0.001
Delay of motor development	2.85	1.08–7.49	0.034
Obesity (BMI >97th percentile)	2.45	1.02–5.90	0.045
**For mild to moderate DP**			
Only bottle feeding	4.68	2.55–8.60	<0.001
Little tummy time when awake	3.22	1.44–7.17	0.004
Obesity (BMI > 97th percentile)	2.29	0.86–6.05	0.096
**For severe DP**			
Only bottle feeding	5.14	2.00–11.10	<0.001
Little tummy time when awake	5.13	1.57–16.73	0.007
Delay of motor development	4.91	1.46–16.51	0.010
Obesity (BMI > 97th percentile)	4.10	1.42–11.90	0.009

### Comparisons According to the Severity of Deformational Plagiocephaly

Compared with controls, infants with mild to moderate DP were more likely to have only been bottle-fed (*p* < 0.001), have little tummy time when awake (*p* = 0.002), and be obese at the time of diagnosis (*p* = 0.021), and those with severe DP were more likely to have only bottle feeding (*p* < 0.001), have little tummy time when awake (*p* = 0.006), be obese at diagnosis (*p* = 0.004), and have a delay in motor development (*p* = 0.012) (Table 1). In the univariate analysis, only bottle feeding was associated with a 4-fold increased risk of mild to moderate DP (aOR = 4.46, 95% CI: 2.48–8.02) and severe DP (aOR = 4.13, 95% CI: 2.02–8.43) compared with controls. Little tummy time when awake was associated with a 3-fold increased risk of mild to moderate DP (aOR = 3.24, 95% CI: 1.52–6.90) and a 4-fold increased risk of severe DP (aOR = 4.63, 95% CI: 1.56–13.75). In addition, obesity at diagnosis increased the risk of mild to moderate DP by 2-fold (aOR = 2.89, 95% CI: 1.17–7.15) and severe DP by 4-fold (aOR = 4.13, 95% CI: 1.59–10.77). The risk of severe DP was increased by 3-fold in infants with delayed motor development (aOR = 3.66, 95% CI: 1.32–10.14). The multivariate analysis for each subgroup showed that only bottle feeding (mild to moderate: aOR = 4.68, 95% CI: 2.55–8.60; severe: aOR = 5.14, 95% CI: 2.00–11.10) and little tummy time when awake (mild to moderate: aOR = 3.22, 95% CI: 1.44–7.17; severe: aOR = 5.13, 95% CI: 1.57–16.73) were significantly associated with both the mild to moderate and severe DP groups. Delay of motor development (aOR = 4.91, 95% CI: 1.46–16.51) and obesity at diagnosis (aOR = 4.10, 95% CI: 1.42–11.90) were significantly associated only with severe DP (Table 2). The comparison between the mild to moderate group and the severe group and the comparison between groups according to brachycephaly accompanying or not showed no significant differences in all the variables.

## Discussion

In this study, we found that only bottle feeding, little tummy time when awake, delay of motor development, and obesity (BMI > 97th percentile) at diagnosis were significantly associated with increased risk of DP. Among these risk factors, delay of motor development and obesity at diagnosis were particularly related to severe DP. Only bottle feeding, little tummy time, and delay of motor development have been identified as risk factors in previous studies, and this study is the first to identify infant obesity (defined by a BMI) as an additional risk factor for DP. This study confirms that infant care relates to DP and its severity; specifically, our findings suggest that infant obesity should be managed together with adjustments in feeding method/position, inclusion of tummy time, and attention to motor development status.

Many studies have demonstrated that unvarying positioning highly contributes to the development of positional preference and positional cranial deformation (2, 6, 17, 18, 23). Risk factors for DP identified in this study included bottle feeding, little tummy time, and delay of motor development. Indeed, only bottle feeding (mostly being fed on the same arm) and little tummy time when awake, along with position while sleeping, have been considered important positioning variables. Factors that have promoted a greater prevalence in bottle feeding include women's increased participation in society, increase in maternal age, increase in the number of mothers, and failure to breast feed exclusively. Both failure to exclusive breast feed and infrequent tummy time can result from lack of education and awareness of proper handling and head positioning and may be contributing factors for high risk of DP in infants (23). Notably, we found an association between delayed motor development and DP, particularly severe DP. Some studies suggest that limited exposure to awake prone positioning is significantly associated with delayed motor development, although the causal relationship between the two factors is difficult to establish (23, 37). Our findings also suggest that unvarying position and body or head positional preference that may have been caused by delayed motor development may be related to DP occurrence, as demonstrated in a previous study (38). Therefore, DP can be a contributor to the elevated risk of delayed motor development, and it is necessary to identify early whether motor development is normal in infants with DP (16, 26, 38). If there is a delay in motor development in infants with DP, early detection of underlying causes and risk factors can aid in implementing interventions, such as physical therapy, and improving neurodevelopment outcomes, thereby preventing severe cranial deformation.

To date, few studies have examined the association between DP and infants' body weight at diagnosis. This study is the first to evaluate the relationship between DP and infant obesity as defined by BMI. Because BMI during the infant period increases until about 6 months after birth and then gradually decreases, the risk of overweight and obesity increases when there is excessive lactation or limited physical activity during the first 6 months (34, 39). In addition, being overweight or obese can make non-varying positioning worse by slowing down and decreasing movement (26). Notably, the first 6 months is also a period when the anterior fontanelle is open and the skull bone grows rapidly; therefore, the risk of cranial deformation is high during that period (40). Our findings suggest a possible mechanism of DP: Overweight or obesity may be the consequence of low physical activity (such as infrequent tummy time), but supine position preference or non-varying position due to overweight or obesity may also contribute to the development of DP, especially severe DP; in other words, DP could be a marker for elevated risk not only for delayed motor development but also for infant obesity. Therefore, it is especially important to assess obesity in infants diagnosed with DP to improve health outcomes due to obesity and prevent the progression to severe DP.

Many studies have demonstrated that rapid or excessive weight gain during infancy is associated with increased risk of childhood or later-life obesity and cardiometabolic dysregulation (26, 41). Traditionally, the clinical guidelines recommend using weight-for-length (WFL) z-score or percentiles to assess body proportionality in children aged ≤2 years. However, growing evidence indicates that BMI may be a more useful index than WFL for assessing adiposity in infants or for predicting the future risk of obesity (26, 39, 42). In this study, both WFL and BMI (z-score and percentile) were identified as indicators of obesity, but only BMI had a significant association with the development and severity of DP. Therefore, it is necessary to use BMI as well as WFL to assess obesity in infants diagnosed with DP so that obesity is not underestimated, and infant obesity and DP are comprehensively evaluated and dually managed.

If DP is not detected in the early stages and left to worsen, facial asymmetry or positional torticollis may also worsen (43, 44), and months of treatment and excessive medical expenses may be required for cranial remodeling (1, 3). In addition, DP is not a minor or purely cosmetic concern, as shown in this study, but is associated with important health problems such as neurodevelopment delays and infant obesity (17, 26). Therefore, there is a need for a multidisciplinary approach and comprehensive management of DP. However, there are concerns that DP is related to various medical specialties, such as pediatrics, neurosurgery, and plastic surgery, and issues other than DP can sometimes be overlooked. Therefore, physicians treating DP should not only pay attention to early detection and intervention for DP but also be able to recognize and manage other health problems associated with DP, such as neurodevelopment and obesity.

This case–control study has several limitations. First, given the retrospective nature of this study, it was difficult to accurately identify the causal relationship between DP and clinical variables and assess the absolute risk of them; in addition, a large number of independent variables compared with the number of objects and the small number of objects of each subgroup might have influenced the results, and the associated factors identified in this study may change depending on different parenting conditions or care habits in other countries or at different time periods (25, 45). Second, our case definition relied on a clinician's objective assessment of the infant head shape, but some controls might have had plagiocephaly, which could have biased OR estimates toward the null. Third, two different measurement methods were used in the diagnosis and severity evaluation of DP. However, given that all infants were diagnosed by a clinician who had expertise and experience in plagiocephaly, such misclassification is unlikely. Fourth, the absence of data on sleep position, positional preference when awake, positioning during feeding, and head shape at birth is also a limitation of our study; however, the absence of these data is unlikely to undermine the significant association between other variables and DP in this study. Fifth, there was no detailed information on tummy time. Thus, the analysis of the variable was simply done in two categories: little tummy time or average tummy time. To clarify the relationship, it is necessary to analyze the tummy time according to age in more detail. Sixth, this study used only AIMS for the assessment of motor development. For a more accurate evaluation of motor development, it is necessary to use other standardized motor development assessment tools, in addition to the AIMS.

In conclusion, our findings confirm that DP risk is positively associated with bottle-only feeding, infrequent tummy time, and delayed development of motor milestones. In addition, this study supports the association between severe DP, a delay in motor development, and infant obesity, which may predispose infants to lower activity levels and less varied positioning. Notably, we identified infant obesity as a new factor related to DP. Our findings suggest the need to assess obesity through BMI levels and to implement preventive practices against further deterioration in infants diagnosed with DP. Further prospective and longitudinal controlled studies involving obesity measurements at multiple time points should be conducted to advance the understanding of DP and its association with infant obesity.

## Data Availability Statement

The raw data supporting the conclusions of this article will be made available by the authors, without undue reservation.

## Ethics Statement

The studies involving human participants were reviewed and approved by the CHA Gangnam Medical Center Institutional Review Board. Written informed consent from the participants' legal guardian/next of kin was not required to participate in this study in accordance with the national legislation and the institutional requirements.

## Author Contributions

E-HK and KK participated in the conception and design of the study. E-HK, KK, JJ, YS, H-SL, SY, NK, and KC performed data acquisition and interpretation. E-HK drafted and edited the article and figures, reviewed the submitted version of the article, and supervised the study. All the authors contributed to the realization of the study and its publication.

## Conflict of Interest

The authors declare that the research was conducted in the absence of any commercial or financial relationships that could be construed as a potential conflict of interest.

## References

[1] Rogers GF Deformational plagiocephaly, brachycephaly, and scaphocephaly. Part I: terminology, diagnosis, and etiopathogenesis. J Craniofac Surg. (2011) 22:9–16. 10.1097/SCS.0b013e3181f6c313 21187783

[2] De Bock F Braun V Renz-Polster H. Deformational plagiocephaly in normal infants: a systematic review of causes and hypotheses. Arch Dis Child. (2017) 102:535–42. 10.1136/archdischild-2016-312018 28104626

[3] Jung BK Yun IS. Diagnosis and treatment of positional plagiocephaly. Arch Craniofac Surg. (2020) 21:80–6. 10.7181/acfs.2020.00059 32380806 PMC7206465

[4] American Academy of Pediatrics. Task force on infant sleep position and sudden infant death syndrome. Changing concepts of sudden infant death syndrome: implications for infant sleeping environment and sleep position. Pediatrics. (2000) 105:650–6. 10.1542/peds.105.3.650 10699127

[5] Mawji A Vollman AR Hatfield J McNeil DA Sauve R. The incidence of positional plagiocephaly: a cohort study. Pediatrics. (2013) 132:298–304. 10.1542/peds.2012-3438 23837184

[6] Ballardini E Sisti M Basaglia N Benedetto M Baldan A Borgna-Pignatti C. Prevalence and characteristics of positional plagiocephaly in healthy full-term infants at 8–12 weeks of life. Eur J Pediatr. (2018) 177:1547–54. 10.1007/s00431-018-3212-0 30030600

[7] Otway C. Plagiocephaly and awareness, prevention and treatment. Community Pract. (2008) 81:38–40. 18497228

[8] Losee JE Mason AC. Deformational plagiocephaly: diagnosis, prevention, and treatment. Clin Plast Surg. (2005) 32:53–64. 10.1016/j.cps.2004.08.003 15636765

[9] Rogers GF. Deformational plagiocephaly, brachycephaly, and scaphocephaly. Part II: prevention and treatment. J Craniofac Surg. (2011) 22:17–23. 10.1097/SCS.0b013e3181f6c342 21187782

[10] Di Chiara A La Rosa E Ramieri V Vellone V Cascone P. Treatment of deformational plagiocephaly with physiotherapy. J Craniofac Surg. (2019) 30:2008–13. 10.1097/SCS.0000000000005665 31232996

[11] Hewitt L Kerr E Stanley RM Okely AD. Tummy time and infant health outcomes: a systematic review. Pediatrics. (2020) 145:e20192168. 10.1542/peds.2019-2168 32371428

[12] Knight S. Positional plagiocephaly/brachycephaly is associated with later cognitive and academic outcomes. J Pediatr. (2019) 210:239–42. 10.1016/j.jpeds.2019.04.042 31234983

[13] Collett BR Wallace ER Kartin D Cunningham ML Speltz ML. Cognitive outcomes and positional plagiocephaly. Pediatrics. (2019) 143:e20182373. 10.1542/peds.2018-2373 30635350 PMC6361360

[14] Leung A Mandrusiak A Watter P Gavranich J Johnston LM. Impact of parent practices of infant positioning on head orientation profile and development of positional plagiocephaly in healthy term infants. Phys Occup Ther Pediatr. (2018) 38:1–14. 10.1080/01942638.2017.1287811 28375778

[15] Hussein MA Woo T Yun IS Park H Kim YO Analysis of the correlation between deformational plagiocephaly and neurodevelopmental delay. J Plast Reconstr Aesthet Surg. (2018) 71:112–7. 10.1016/j.bjps.2017.08.015 28958569

[16] Wittmeier K Mulder K. Time to revisit tummy time: a commentary on plagiocephaly and development. Paediatr Child Health. (2017) 22:159–61. 10.1093/pch/pxx046 29479204 PMC5804977

[17] Martiniuk AL Vujovich-Dunn C Park M Yu W Lucas BR. Plagiocephaly and developmental delay: a systematic review. J Dev Behav Pediatr. (2017) 38:67–78. 10.1097/DBP.0000000000000376 28009719

[18] Bialocerkowski AE Vladusic SL Wei Ng C. Prevalence, risk factors, and natural history of positional plagiocephaly: a systematic review. Dev Med Child Neurol. (2008) 50:577–86. 10.1111/j.1469-8749.2008.03029.x 18754894

[19] Mawji A Vollman AR Fung T Hatfield J McNeil DA Sauve R. Risk factors for positional plagiocephaly and appropriate time frames for prevention messaging. Paediatr Child Health. (2014) 19:423–7. 10.1093/pch/19.8.423 25382999 PMC4220526

[20] Ahluwalia R Kiely C Foster J Gannon S Wiseman AL Shannon CN. Positional posterior plagiocephaly: a single-center review. J Neurosurg Pediatr. (2020). 10.3171/2019.12.PEDS19651. [Epub ahead of print]. 32005011

[21] Habal MB Castelano C Hemkes N Scheuerle J Guilford AM. In search of causative factors of deformational plagiocephaly. J Craniofac Surg. (2004) 15:835–41. 10.1097/00001665-200409000-00025 15346027

[22] Glasgow TS Siddiqi F Hoff C Young PC. Deformational plagiocephaly: development of an objective measure and determination of its prevalence in primary care. J Craniofac Surg. (2007) 18:85–92. 10.1097/01.scs.0000244919.69264.bf 17251842

[23] van Vlimmeren LA van der Graaf Y Boere-Boonekamp MM L'Hoir MP Helders PJ Engelbert RH. Risk factors for deformational plagiocephaly at birth and at 7 weeks of age: a prospective cohort study. Pediatrics. (2007) 119:e408–18. 10.1542/peds.2006-2012 17272603

[24] Colson ER Willinger M Rybin D Heeren T Smith LA Lister G. Trends and factors associated with infant bed sharing, 1993–2010: the National Infant Sleep Position Study. JAMA Pediatr. (2013) 167:1032–7. 10.1001/jamapediatrics.2013.2560 24080961 PMC3903787

[25] McKinney CM Cunningham ML Holt VL Leroux B Starr JR. Characteristics of 2733 cases diagnosed with deformational plagiocephaly and changes in risk factors over time. Cleft Palate Craniofac J. (2008) 45:208–16. 10.1597/06-227.1 18333652

[26] Koren A Kahn-D'angelo L Reece SM Gore R. Examining childhood obesity from infancy: the relationship between tummy time, infant BMI-z, weight gain, and motor development-an exploratory study. J Pediatr Health Care. (2019) 33:80–91. 10.1016/j.pedhc.2018.06.006 30131199

[27] Loveday BP de Chalain TB Active counterpositioning or orthotic device to treat positional plagiocephaly? J Craniofac Surg. (2001) 12:308–13. 10.1097/00001665-200107000-00003 11482615

[28] Holowka MA Reisner A Giavedoni B Lombardo JR Coulter C. Plagiocephaly severity scale to aid in clinical treatment recommendations. J Craniofac Surg. (2017) 28:717–22. 10.1097/SCS.0000000000003520 28468155

[29] Cevik S Isik S Ozkilic A. The role of age on helmet therapy in deformational plagiocephaly and asymmetric brachycephaly. Childs Nerv Syst. (2020) 36:803–10. 10.1007/s00381-019-04354-2 31482314

[30] Graham T Millay K Wang J Adams-Huet B O'Briant E Oldham M. Significant factors in cranial remolding orthotic treatment of asymmetrical brachycephaly. J Clin Med. (2020) 9:1027. 10.3390/jcm9041027 32260587 PMC7231243

[31] WHO Anthro for personal computers, version 3. Software for assessing growth and development of the world's children. (2009). Available online at: http://www.who.int/childgrowth/software/en/ (accessed June 1, 2020).

[32] Group WHOMGRS. WHO Child Growth Standards based on length/height, weight and age. Acta Paediatr Suppl. (2006) 450:76–85. 10.1111/j.1651-2227.2006.tb02378.x 16817681

[33] Woo JG Daniels SR. Assessment of body mass index in infancy: it is time to revise our guidelines. J Pediatr. (2019) 204:10–1. 10.1016/j.jpeds.2018.09.025 30297288

[34] Sun J Nwaru BI Hua J Li X Wu Z. Infant BMI peak as a predictor of overweight and obesity at age 2 years in a Chinese community-based cohort. BMJ Open. (2017) 7:e015122. 10.1136/bmjopen-2016-015122 28988164 PMC5640041

[35] Fuentefria RDN Silveira RC Procianoy RS. Motor development of preterm infants assessed by the Alberta Infant Motor Scale: systematic review article. J Pediatr (Rio J). (2017) 93:328–42. 10.1016/j.jped.2017.03.003 28506665

[36] Stellwagen L Hubbard E Chambers C Jones KL. Torticollis, facial asymmetry and plagiocephaly in normal newborns. Arch Dis Child. (2008) 93:827–31. 10.1136/adc.2007.124123 18381343

[37] Majnemer A Barr RG. Influence of supine sleep positioning on early motor milestone acquisition. Dev Med Child Neurol. (2005) 47:370–6. 10.1017/S0012162205000733 15934485

[38] Speltz ML Collett BR Stott-Miller M Starr JR Heike C Wolfram-Aduan AM. Case-control study of neurodevelopment in deformational plagiocephaly. Pediatrics. (2010) 125:e537–42. 10.1542/peds.2009-0052 20156894 PMC3392083

[39] Slining MM Herring AH Popkin BM Mayer-Davis EJ Adair LS. Infant BMI trajectories are associated with young adult body composition. J Dev Orig Health Dis. (2013) 4:56–68. 10.1017/S2040174412000554 24040489 PMC3769801

[40] Hutchison BL Hutchison LA Thompson JM Mitchell EA. Plagiocephaly and brachycephaly in the first two years of life: a prospective cohort study. Pediatrics. (2004) 114:970–80. 10.1542/peds.2003-0668-F 15466093

[41] Benjamin Neelon SE Schou Andersen C Schmidt Morgen C Kamper-Jorgensen M Oken E Gillman MW. Early child care and obesity at 12 months of age in the Danish National Birth Cohort. Int J Obes (Lond). (2015) 39:33–8. 10.1038/ijo.2014.173 25233894 PMC4286493

[42] Roy SM Spivack JG Faith MS Chesi A Mitchell JA Kelly A Infant BMI or weight-for-length and obesity risk in early childhood. Pediatrics. (2016) 137:e20153492 10.1542/peds.2015-3492 27244803 PMC4845873

[43] Hummel P Fortado D. Impacting infant head shapes. Adv Neonatal Care. (2005) 5:329–40. 10.1016/j.adnc.2005.08.009 16338671

[44] van Vlimmeren LA Helders PJ van Adrichem LN Engelbert RH. Torticollis and plagiocephaly in infancy: therapeutic strategies. Pediatr Rehabil. (2006) 9:40–6. 10.1080/13638490500037904 16352505

[45] McKinney CM Cunningham ML Holt VL Leroux B Starr JR. A case-control study of infant, maternal and perinatal characteristics associated with deformational plagiocephaly. Paediatr Perinat Epidemiol. (2009) 23:332–45. 10.1111/j.1365-3016.2009.01038.x 19523080

